# Infants' Peripheral Blood Lymphocyte Composition Reflects Both Maternal and Post-Natal Infection with *Plasmodium falciparum*

**DOI:** 10.1371/journal.pone.0139606

**Published:** 2015-11-18

**Authors:** Odilon Nouatin, Komi Gbédandé, Samad Ibitokou, Bertin Vianou, Parfait Houngbegnon, Sem Ezinmegnon, Sophie Borgella, Carine Akplogan, Gilles Cottrell, Stefania Varani, Achille Massougbodji, Kabirou Moutairou, Marita Troye-Blomberg, Philippe Deloron, Adrian J. F. Luty, Nadine Fievet

**Affiliations:** 1 Centre d’Etude et de Recherche sur le Paludisme Associé à la Grossesse et à l’Enfance (CERPAGE), Faculté des Sciences de la Santé, Université d’Abomey-Calavi, Cotonou, Benin; 2 Département de Biochimie et de Biologie Cellulaire, Faculté des Sciences et Techniques, Université d’Abomey-Calavi, Cotonou, Bénin; 3 Institut de Recherche pour le Développement, MERIT UMR D216 Mère et enfant face aux infections tropicales, Paris, France; 4 PRES Sorbonne Paris Cité, Université Paris Descartes, Faculté de Pharmacie, Paris, France; 5 Unit of Microbiology, Department of Diagnostic, Experimental and Specialty Medicine, University of Bologna, Bologna, Italy; 6 Department of Molecular Biosciences, the Wenner-Gren Institute, Stockholm University, Stockholm, Sweden; 7 Department of Medical Microbiology, Radboud University Nijmegen Medical Centre, Nijmegen, The Netherlands; Université Pierre et Marie Curie, FRANCE

## Abstract

Maternal parasitoses modulate fetal immune development, manifesting as altered cellular immunological activity in cord blood that may be linked to enhanced susceptibility to infections in early life. *Plasmodium falciparum* typifies such infections, with distinct placental infection-related changes in cord blood exemplified by expanded populations of parasite antigen-specific regulatory T cells. Here we addressed whether such early-onset cellular immunological alterations persist through infancy. Specifically, in order to assess the potential impacts of *P*. *falciparum* infections either during pregnancy or during infancy, we quantified lymphocyte subsets in cord blood and in infants' peripheral blood during the first year of life. The principal age-related changes observed, independent of infection status, concerned decreases in the frequencies of CD4^+^, NK^dim^ and NKT cells, whilst CD8^+^, Treg and Teff cells' frequencies increased from birth to 12 months of age. *P*. *falciparum* infections present at delivery, but not those earlier in gestation, were associated with increased frequencies of Treg and CD8^+^ T cells but fewer CD4^+^ and NKT cells during infancy, thus accentuating the observed age-related patterns. Overall, *P*. *falciparum* infections arising during infancy were associated with a reversal of the trends associated with maternal infection i.e. with more CD4^+^ cells, with fewer Treg and CD8^+^ cells. We conclude that maternal *P*. *falciparum* infection at delivery has significant and, in some cases, year-long effects on the composition of infants' peripheral blood lymphocyte populations. Those effects are superimposed on separate and independent age- as well as infant infection-related alterations that, respectively, either match or run counter to them.

## Introduction

Infectious diseases during pregnancy affect infants’ responses to vaccination [1, 2] their susceptibility to postnatal infection [3] and their development of immunopathological disorders such as allergy [4]. These detrimental and long-lasting outcomes are the result of exposures *in utero* that alter foetal immune responses. Examples of diseases known to have such effects include the malaria parasite, *Plasmodium falciparum*, *Trypanosoma cruzi*, the cause of Chagas’ disease, *Schistosoma* spp that cause bilharzia, and *Wuchereria bancrofti*, one of the species of nematode worm responsible for filariasis [5–8].

Malaria due to *P*. *falciparum* causes an estimated 584 000 deaths per year in African children and is also responsible for severe morbidity in pregnant women (WHO 2014). Sequestration and massive accumulation of infected erythrocytes in the placenta is the primary pathological phenomenon that characterizes pregnancy-associated malaria (PAM) [9]. PAM leads to a higher risk of maternal and foetal anemia, intra-uterine growth retardation, low birth weight and prematurity [10].

It is now well-established that *P*. *falciparum* infection of the mother during pregnancy will also impact subsequent weight and growth development in early life [11, 12]. PAM also influences susceptibility to malaria [13, 14] and to other infections [15] in early life, suggesting long-lasting post-natal effects of PAM. The immunological mechanisms that establish and maintain what has been termed the ‘tolerant phenotype’ phenomenon with respect to PAM and malaria in infancy are not well understood [1]. That PAM does demonstrably affect immune functions in the newborn has nevertheless been reported in several studies, characterized by

1. modulation of immunological responses at birth (in cord blood) as well as during infancy and childhood in those born to mothers with placental infection at delivery [16–18];
2. the presence of parasite antigen-specific memory and regulatory T cells in cord blood from exposed children [19–21],
3. larger populations of T regulatory CD4^+^ cells (Treg) in cord blood from offspring of women with PAM when compared with those of uninfected women [19, 22].

During childhood, the proportion of CD4^+^ and CD8^+^ T cells increases, attaining a profile resembling that of adults by 5 years of age [23]). CD4^+^ T helper (Th) cells are mainly involved in the activation of other immune cells to construct an immune response, while CD8^+^ T cells actively eliminate infected cells through their cytotoxic properties. An increased proportion of CD4^-^ T cells (comprising mostly CD8^+^ cells) before the age of 5 years, whilst the proportion of CD4^+^ T cells was stable during this period, has also been reported [24]. These fluctuations in the relative numbers of CD4^+^ and CD8^+^ cells during early life may be a reflection of a continuous redistribution among these subsets throughout life [25].

Treg suppress antigen-specific responses *in vivo* and *in vitro* and are involved in the control of autoimmune responses. The frequency of Treg has been reported to increase in *P*. *falciparum*-infected adults with a dose-dependent effect of parasite load [26–29]. During foetal and early life, the development of Treg is also crucial to suppress foetal anti-maternal immunity and in the maintenance of foetal self-tolerance [30]. These cells produce large amounts of IL-10 [31] [32], and inhibit the production of Th1 cytokines in monocytes, macrophages, T cells and natural killer (NK) cells. Treg have also been shown to play a role in the downmodulation of NK cells’ activities [33].

In humans, two distinct NK cell populations have been identified based on differential CD16 and CD56 expression. The majority of NK cells in peripheral blood are NK^dim^ (CD56^low^ CD16^high^) with cytotoxicity as their main effector function. The minor population is defined as NK^bright^ (CD56^high^ CD16^low^) and favours cytokine production [34]. It has been shown that NK cells from cord blood are phenotypically and functionally mature [35]’ and able to produce interferon-γ (IFN-γ) and proliferate in response to IL-2, in a manner comparable to those of adults. NK cells purportedly play an important role in early immunity against malaria, being among the first cells in peripheral blood to produce protective IFN-γ in response to *P*. *falciparum*-infected erythrocytes [36, 37]. Further, IFN-γ-producing NK cells in the placenta have been reported to exert a protective role against PAM [38]. The expansion of a subset of T cells expressing NK cell markers, NKT cells [39], has been reported in peripheral blood during severe *P*. *falciparum* infection [40] and in the livers of mice in a rodent model of malaria infection, where these cells were also associated with anti-parasitic activity [41]. NKT cells are normally present in very low numbers in peripheral blood, but they are activated *in utero* [42], likely contributing to the control of foetal Th2 responses [43].

Thus, different cellular components of the innate immune system, including Treg, NK and NKT cells, are known to be associated with putatively protective responses against *P*. *falciparum*. However, prospective studies of the impact of PAM on the profile and function of these cells in infants have yet to be reported. The aim of the study described here was thus to determine the phenotypic profiles of different circulating lymphocyte populations of infants born to mothers with or without PAM and to evaluate prospectively whether *P*. *falciparum* infection of the mother during pregnancy or of the infant would affect the profiles of these cells during the first year of life.

## Materials and Methods

### Ethics statement

The study was approved by the ethics committees of the Faculty of Health Sciences (University of Abomey-Calavi, Benin) and of the Research Institute for Development (IRD) in France. Written informed consent was obtained from all women or guardians on behalf of the minors/children included in the study.

### Study design

The STOPPAM project (Strategies to Prevent Pregnancy Associated Malaria) was carried out in Benin and Tanzania between November 2008 and April 2011. Detailed descriptions of the study sites, demographics of study populations, study design and conduct have been published previously [44]. In Benin, the study took place in a high malaria transmission area located 70 km west of the commercial capital, Cotonou. The entomological inoculation rate estimated in a neighbouring area is 35–60 infective bites per person per year [45]. Three dispensaries were involved in enrolling pregnant women with a gestational age less than 24 weeks: Come, Akodeha and Ouedeme Pedah. Women enrolled in the study had monthly clinical and parasitological follow-up assessments until delivery, and received intermittent preventive treatment with sulphadoxine-pyrimethamine during scheduled antenatal visits, according to national policy in Benin. Women with a diagnosis of clinical malaria (temperature ≥37.5°C and positive malaria rapid diagnostic test, RDT) received a treatment regimen of quinine. In case of clinical symptoms between antenatal visits (ANV), women were encouraged to attend the maternity clinic to receive care.

**Fig 1:**
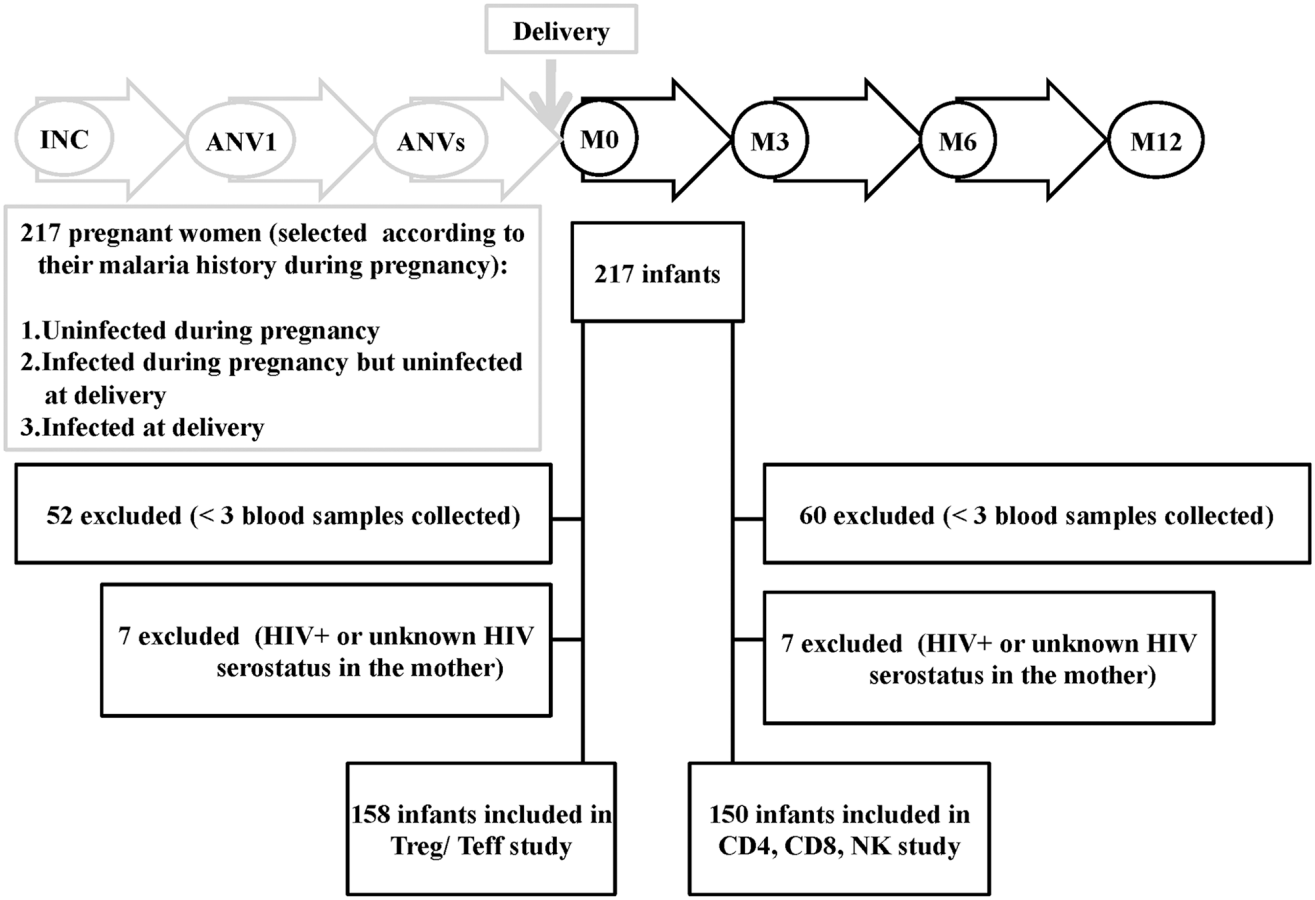
Flow diagram of birth cohort study. 217 pregnant women were enrolled under 24 weeks of gestation and their infants were longitudinally followed-up from birth to 12 months of age. For the Treg/Teff part: 59 newborns were excluded (52 newborns < 3 blood samples collected and 7 newborns HIV+ or unknown HIV serostatus in the mother). Data from 158 infants were included for analyses. For the CD4, CD8, NK part: 67 newborns were excluded (60 newborns < 3 blood samples collected and 7 newborns HIV^+^ or unknown HIV serostatus in the mother). Data from 150 infants were included for analyses.

As part of the STOPPAM project, 217 pregnant women were selected on the basis of their *P*. *falciparum* infection histories (uninfected during pregnancy, n = 99; infected during pregnancy but uninfected at delivery, n = 71; infected at delivery, n = 47); following delivery, the infants of these mothers were followed-up every two weeks from birth to 12 months of age. The follow-up of infants included routine tests to detect *P*. *falciparum*, clinical assessments and growth measurements, details of all of which have been published previously (Fig 1) [46]. To perform immunological studies, 10 ml of cord blood were collected at delivery, while 2 ml peripheral blood samples were obtained at 3, 6 and 12 months of age. Samples were processed at the laboratory of the Research Center for Malaria in Pregnancy and Infancy (CERPAGE). Infants for whom fewer than 3 blood samples were collected during the follow-up period were excluded from the study as well as infants whose mothers tested positive for HIV or had an unknown HIV serostatus.

### *P*. *falciparum* infection status

Clinical and parasitological assessments were performed at each ANV in mothers and during the follow-up of infants. Parasites were detected by thick blood smear (TBS). Briefly, smears were prepared, stained with Giemsa and examined by two experienced laboratory technicians for the presence and density of parasites. Smears were considered negative if no asexual stage *Plasmodium* parasite was detected by counting 500 leucocytes. Parasites were counted against 200 leukocytes and parasite density was estimated assuming 8000 leukocytes/μl of blood. At delivery, TBS were made from peripheral, placental and cord blood samples. Infection with *P*. *falciparum* in the mothers at delivery was defined by the presence of parasites in placental and/or maternal peripheral blood.

### Immunophenotyping

The monoclonal antibodies (mAb) used for T- and NK-cell labeling were the following: anti-CD8-FITC, anti-CD4-PerCP, anti-CD3-APC, anti-CD56-PE antibodies (all BD Biosciences). For Treg and CD4^+^ T effector cell (Teff) labeling anti-CD4-PerCP, anti-CD25-FITC, anti-CD127-PE and anti-Foxp3-APC monoclonal antibodies were used (BD Biosciences and eBioscience).

In a first step, 5μl of FcR blocking reagent (Miltenyi Biotec, Cologne, Germany) were added to 100μl of whole blood *ex vivo* and incubated for 15 min at 4°C. Cells were then specifically labeled by adding 5μl of each mAb, lightly vortexed and then incubated for 30 min at 4°C in the dark. Two ml of Facs Lysing buffer (BD Biosciences) were then added and cells incubated for a further 15 min at room temperature, in the dark. After spin-washing twice with PBS 3% FCS, at least 200,000 cells were then acquired on a flow cytometer (BD FacsCalibur).

Treg and Teff labeling was performed according to the manufacterer’s recommendation (eBioscience). Briefly, 1 ml whole blood *ex vivo* was washed with PBS-3% Foetal Bovin Serum (FBS). After washing, 8 μl of anti-CD25-FITC, 6 μl of anti-CD127-PE and 6μl of anti-CD4-PerCP antibodies were added. Cells were incubated for 25 min at 4°C. Red blood cells were then lysed by adding Facs Lysing buffer and cells incubated for 15 min in the dark. After spin-washing with PBS, cells were incubated overnight with Fix-Perm buffer (eBioscience), then washed with Perm-Wash Buffer (eBioscience). Cells were then incubated in Perm-Wash Buffer with 2% rat serum (eBioscience) for 15 minutes in the dark, followed by the addition of 10μl of anti-Foxp3-APC and incubated for a further 15 minutes in the dark. After two spin-washes with Perm-Wash Buffer and PBS 3% FCS, at least 1,000,000 cells were acquired.

Treg (CD4^+^CD25^+^CD127^-^) and Teff (CD4^+^CD25^+^CD127^+^) cells were gated from the whole lymphocyte population (S1 Fig). CD4^+^ and CD8^+^ were gated on CD3^+^ cells, whilst NK cells were characterized as CD3^-^CD56^+^ cells and NKT cells were defined as CD3^+^CD56^+^ cells. For the purposes of our study, NK^dim^ cells were defined as CD3^-^ cells with weak expression of CD56, whereas NK^bright^ cells were also CD3^-^ but with strong expression of CD56. Levels of expression of Foxp3 were evaluated with a relative value (RV) corresponding to the MFI of Foxp3 in the Treg (or Teff) divided by the MFI of FoxP3 in the naïve T cell population (CD4^+^CD25^-^CD127^+^)

### Statistical analysis

The association between T- and NK-cell frequencies and infant age was investigated by using analyses of variances for repeated measures.

To examine the contribution of PAM and/or infant plasmodial infection to T- and NK-cell frequencies, the first set of analyses proceeded as follows: for each phenotype frequency we built a multivariate linear mixed model (LMM) that takes into account the correlation between repeated measurements as well as other potential confounders in the relationship between infection and the phenotype frequency. We proceeded in two steps; first a univariate model investigated the association between baseline characteristics and T-/NK-cell frequencies. Baseline characteristics were gravidity, maternal anaemia (Hb < 11 g/dl), prematurity (<37 weeks), low birth weight (< 2500 g) and infant gender. Variables showing a p-value < 0.2 were selected for the multivariate model (second step).

We built 3 variables related to maternal plasmodial infections as follows: (i) infection before the third trimester of pregnancy, (ii) infection during the third trimester of pregnancy but more than 10 days before delivery, (iii) infection from 10 days prior to delivery up to and including delivery. Designation of the latter group was based on the premise that infections detected during an emergency visit occurring 10 days or less before delivery, and therefore treated, were too close in time to delivery to be separable from it, and because most of those concerned were also found to be infected at delivery.

We also built 3 variables related to infections during infancy as follows: (i) those occurring before three months of age, (ii) those occurring between 3 and 6 months of age, (iii) those occurring between 6 and 12 months of age. All the variables related to *P*. *falciparum* infection were included in the multivariate model with the baseline characteristics selected at the univariate step. The final multivariate model retained all the variables related to *P*. *falciparum* infection along with the baseline characteristics showing significance at the 0.05 level.

To graphically illustrate the predicted effect of maternal infection on the phenotype frequencies of infants, we then computed the mean predicted phenotype frequencies of infants born from uninfected mothers at each time-point, as well as those of the same infants if they were born to an infected mother.

In a second set of analyses, the impact of infant *P*. *falciparum* infection on their T-/NK-cell frequencies was examined through three questions. Does infant *P*. *falciparum* infection before 3 months of age have a significant effect on the T-/NK-cell responses at 3 months? Does infant *P*. *falciparum* infection between 3 and 6 months of age have a significant effect on their T-/NK-cell responses at 6 months? Does infant *P*. *falciparum* infection between 6 and 12 months of age have a significant effect on the T-cell/NK-cell responses at 12 months? To answer each question we employed three linear regression models (at 3 months, 6 months and 12 months). The final models were obtained after adjusting for maternal *P*. *falciparum* infection, gravidity, sex, prematurity and LBW.

In a third set of analyses we aimed to identify whether T-/NK-cell frequencies could affect the occurrence of infection during the ensuing months. To achieve this aim we employed a logistic mixed model where the dependent variable was the time-dependent binary variable “occurrence of infection between 2 consecutive measurement of the T-/NK-cell frequencies” (i.e. infection arising before 3 months of age, infection occurring between 3 and 6 months of age, and infection occurring between 7 and 12 months of age). This dependent variable was regressed on the T-/NK-cell frequencies in 2 different ways: in a first model we determined whether phenotype frequencies at birth were associated with the occurrence of infection during the first 12 months of age; in this model the dependent “infection occurence” variable was regressed on the T-/NK-cell frequencies in cord blood. In a second model, we built a time-dependent covariate “T-cell/NK-cell frequencies at the previous measurement”, so that, for infection occurring prior to 3 months of age, we considered the T-/NK-cell frequencies in cord blood cells, for occurrence of infection between 3 and 6 months of age we considered T-/NK-cell frequencies in blood cells obtained at 3 months of age and for occurrence of infection between 7 and 12 months of age we considered T-/NK-cell frequencies in blood cells obtained at 6 months of age. As in the previous analyses, potential confounders were first analyzed in a univariate model and then in a multivariate model. The models allowed estimation of the adjusted values of the phenotype frequencies (for any set of covariates) of all infants at each time-point.

Statistical significance in all multivariate analyses was considered if *p* < 0.05. All analyses were performed using the R statistical package (R Development Core Team; R Foundation for Statistical Computing, Vienna, Austria; http://www.R-project.org) and graphs made with graphPad (Prism 5.0).

## Results

### Characteristics of the study population

Between November 2008 and April 2011, 217 mother/infant pairs were enrolled in the study (Fig 1) but of these 59 were excluded due to insufficient numbers of blood samples (52) and HIV sero-status (7 either HIV+ or unknown). The characteristics of the 158 pairs retained for the analysis presented here are presented in Table 1. Women had a mean age of 26.8 years (95% CI 25.9–27.7), whilst 27 (17.1%) were primigravidae and 18 (11.4%) had anemia (Hb<11g/dL) at delivery.

**Table 1:** Characteristics of the study population (n = 158 mother/infant pairs).

Mother		n (%)
Age (years)	≤ 20	34 (21.5)
	21–25	45 (28.5)
	26–30	41 (26)
	≥ 31	38 (24)
Gravidity status	Primigravidae	27 (17.1)
	Multigravidae	131 (82.9)
Anemia at delivery (Hb<11g/dl)		18 (11.4)
Infected before 3^rd^ trimester of pregnancy		50 (31.6)
Infected during 3^rd^ trimester of pregnancy up until 10 days before delivery		32 (20.3)
Infected 10 days before or at delivery		38 (24.1)
No sign of infection		38 (24.1)
Infected placenta		29 (18.8)
Infant		
Sex	Female	76 (48.1)
Prematurity (gestational age ≤ 37 weeks)		12 (7.6)
Residence location	Rural	44 (27.8)
	Semi-rural	114 (72.2)
Low birth weight (< 2500 g)		19 (12)
Infected before 3 months of age		6 (3.8)
Infected between 3 and 6 months of age		20 (12.7)
Infected between 6 and 12 months of age		47 (29.7)
No sign of infection		85 (53,9)

Infections before the third trimester of pregnancy were detected in 50 (32%) women, whilst 32 (20%) were infected during the third trimester of pregnancy, and in 38 (24%) women infections occurred within 10 days of or at delivery. The latter group of women infected 10 days before or at delivery comprised women infected at delivery and women shown to be infected close to delivery when attending an emergency visit; most of the women belonging to the latter group were also found to be infected at delivery. Babies' mean birth weight was 3053g (2987–3119).

### Variations in T- and NK cell frequencies according to age in the first year of life

**Fig 2:**
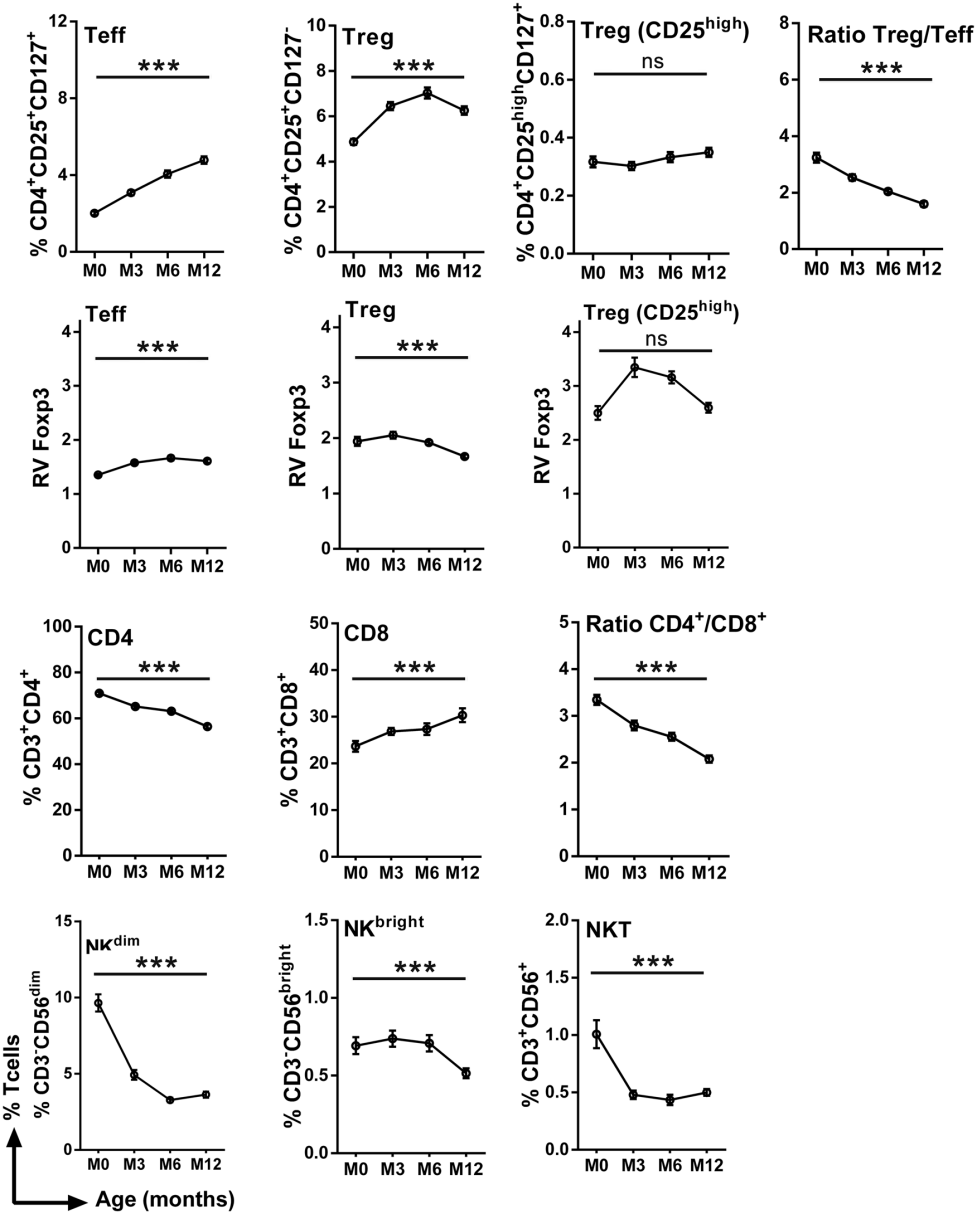
Frequencies of cell subsets in infants' peripheral blood during the first year of life. Curves include dots representing mean with SEM (standard error of the mean). The statistical significance of differences indicated refers to analyses of variances for repeated measures. ***, p <0.001.

There was a significant effect of infant age on the T lymphocyte profiles during the first year of life. Thus the frequency of CD4^+^ cells was higher at birth and decreased with age, while the frequency of CD8^+^ cells was lower at birth and increased with age; the CD4^+^/CD8^+^ ratio consequently decreased with age (Fig 2). Despite the overall decline in CD4^+^ cells, the frequencies of Treg and of Teff increased between 0 and 12 months of age, although the Treg/Teff ratio decreased with infant’s age, suggesting that Teff increased more rapidly than Treg (Fig 2). Foxp3 was detected in Treg and to a lesser extent in Teff, with a clearly higher level of expression in Treg. The subset of Treg with the highest levels of expression of CD25 expressed the highest levels of Foxp3 (Fig 2), a key transcription factor that is required for development, maintenance, and function of these cells [47], suggesting a Treg cell subpopulation with specific immune function. With increasing age, the relative expression of Foxp3 increased in Teff and decreased Treg. In the CD25^high^ subpopulation of Treg the relative level of Foxp3 expression increased from birth to 6 months of age but declined thereafter (Fig 2). The frequencies of all NK cell subpopulations decreased with age (Fig 2). With the exception of the CD25^high^ subpopulation of Treg all of these age-dependent variations were shown to be statistically significant (p<0.001) firstly in analyses of variance for repeated measures (Fig 2), and subsequently in multivariate analyses (Tables 2 and 3).

**Table 2:** Multivariate (LMM) analyses of alterations in circulating T- and NK-cell subset frequencies in peripheral blood as a function of infants’ age and of *P*. *falciparum* infection at delivery.

			Treg			Teff			Treg (CD25^high^)			Ratio Treg/Teff			R FoxP3 in Treg			R FoxP3 in Teff			R FoxP3 in Treg (CD25^high^)		
			Regression		p	Regression		p	Regression		p	Regression		p	Regression		p	Regression		p	Regression		p
			coef, (SD)^D^			coef, (SD)			coef, (SD)			coef, (SD)			coef, (SD)			coef, (SD)			coef, (SD)		
Infant age^A^	3 months		1,45	0,26	<0.01	0,97	0,22	<0.01	-0,02	0,03	ns	-0,64	0,18	<0.01	0,16	0,09	ns	0,23	0,05	<0.01	0,92	0,21	<0.01
	6 months		2,18	0,26	<0.01	1,99	0,23	<0.01	0,00	0,03	ns	-1,10	0,18	<0.01	-0,03	0,10	ns	0,32	0,05	<0.01	0,74	0,21	<0.01
	12 months		1,35	0,26	<0.01	2,73	0,22	<0.01	0,03	0,02	ns	-1,64	0,18	<0.01	-0,27	0,09	<0.01	0,23	0,05	<0.01	0,09	0,21	ns
*P*. *falciparum* maternal infection	at delivery^B^	& 0 M	0,47	0,43	ns	-0,11	0,36	ns	-0,01	0,04	ns	0,31	0,29	ns	0,18	0,13	ns	-0,04	0,07	ns	0,48	0,31	ns
		& 3 M	1,10	0,44	<0.05	0,44	0,37	ns	0,04	0,04	ns	0,07	0,29	ns	-0,04	0,14	ns	-0,07	0,08	ns	0,17	0,31	ns
		& 6 M	0,38	0,44	ns	0,05	0,37	ns	0,04	0,04	ns	-0,03	0,29	ns	0,20	0,14	ns	-0,09	0,08	ns	0,13	0,31	ns
		& 12 M	0,52	0,43	ns	0,02	0,36	ns	0,00	0,04	ns	0,27	0,29	ns	0,16	0,14	ns	0,08	0,08	ns	0,47	0,30	ns

^A^ The reference values used for comparison are those recorded in cord blood (M0);

^B^ denotes the influence of infection at delivery/≤ 10 days prior to delivery on neonatal/infant responses measured at designated time-points; M0: cord blood, M3, M6, M12: blood drawn at 3, 6 & 12 months of age;

^C^ denotes the influence of infection at time-points in the infant on the compostion of neonatal/infant peripheral blood lymphocyte subsets.

^D^ Positive/negative coefficients indicate cell subset frequencies above/below control (uninfected) levels; SD: standard deviation. All the data were adjusted on *P*. *falciparum* infection history of mother, gravidity, infant age, low birth weight

**Table 3:** Multivariate (LMM) analyses of alterations in circulating T- and NK-cell subset frequencies in peripheral blood as a function of infants’ age and of *P*. *falciparum* infection at delivery.

			CD4			CD8			Ratio CD4/CD8			NKT			NK^dim^			NK^bright^		
			Regression coef, (SD)		p	Regression coef, (SD)		p	Regression coef, (SD)		p	Regression coef, (SD)		p	Regression coef, (SD)		p	Regression coef, (SD)		p
Infant age^A^	3 months		-0,08	0,02	<0.01	0,11	0,03	<0.01	-0,55	0,12	<0.01	-0,70	0,09	<0.01	-0,48	0,09	<0.01	0,36	0,15	<0.05
	6 months		-0,11	0,02	<0.01	0,15	0,03	<0.01	-0,79	0,12	<0.01	-0,79	0,09	<0.01	-0,84	0,09	<0.01	0,34	0,15	<0.05
	12 months		-0,25	0,02	<0.01	0,28	0,03	<0.01	-1,34	0,12	<0.01	-0,54	0,09	<0.01	-0,75	0,10	<0.01	0,01	0,15	ns
*P*. *falciparum* maternal infection	at delivery^B^	& 0 M	-0,02	0,03	ns	0,15	0,06	<0.05	-0,61	0,21	<0.01	-0,54	0,14	<0.01	0,44	0,14	<0.01	0,31	0,22	ns
		& 3 M	-0,08	0,03	<0.05	0,17	0,06	<0.05	-0,65	0,22	<0.01	-0,18	0,14	ns	-0,28	0,14	<0.05	-0,24	0,23	ns
		& 6 M	-0,07	0,03	<0.05	0,14	0,06	<0.05	-0,55	0,22	<0.05	-0,28	0,14	<0.05	0,00	0,14	ns	-0,11	0,22	ns
		& 12 M	-0,01	0,04	ns	0,05	0,06	ns	-0,25	0,22	ns	-0,31	0,14	<0.05	-0,09	0,15	ns	0,30	0,23	ns

^A^ The reference values used for comparison are those recorded in cord blood (M0);

^B^ denotes the influence of infection at delivery/≤ 10 days prior to delivery on neonatal/infant responses measured at designated time-points; M0: cord blood, M3, M6, M12: blood drawn at 3, 6 & 12 months of age;

^C^ denotes the influence of infection at time-points in the infant on the compostion of neonatal/infant peripheral blood lymphocyte subsets.

^D^ Positive/negative coefficients indicate cell subset frequencies above/below control (uninfected) levels; SD: standard deviation. All the data were adjusted on *P*. *falciparum* infection history of mother, gravidity, infant age, low birth weight

Thus, we observed variations in the frequency of T- and NK-cell subsets within the first year of life, with an overall decrease of CD4, NK^dim^ and NKT cells and an increase of CD8, Treg and Teff over time.

### Maternal *P*. *falciparum* infection at delivery influences the immune cell profile of infants

Next, we examined whether maternal *P*. *falciparum* infection could influence the frequencies of lymphocytes in the offspring.

**Fig 3:**
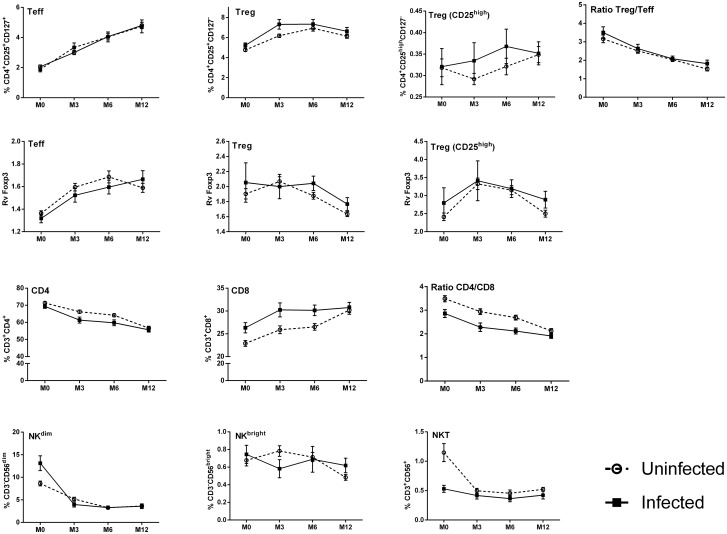
Frequencies of cell subsets in infants' peripheral blood over time segregated on the basis of presence or absence of maternal infection at delivery. (white for negative and hatched for maternal infection). Curves include dots representing mean with SEM (standard error of the mean).

Univariate analyses showed that *P*. *falciparum* infection before the third trimester of pregnancy was associated only with a decreased frequency of NK^dim^ in infants (data not shown), whilst infection during the third trimester of pregnancy had no effect on any parameter (data not shown). In contrast, maternal *P*. *falciparum* infection at delivery was associated with several alterations in infant’s lymphocyte populations (S1 Table, Fig 3). Most notable were the increased frequencies of both Treg and of CD8^+^ cells throughout infancy but, conversely, reduced frequencies of both CD4^+^ and NKT cells (Fig 3). Further, maternal infection at delivery was associated with an increased frequency of infant NK^dim^ cells at birth, but no effect was seen on NK^bright^ frequencies.

**Fig 4:**
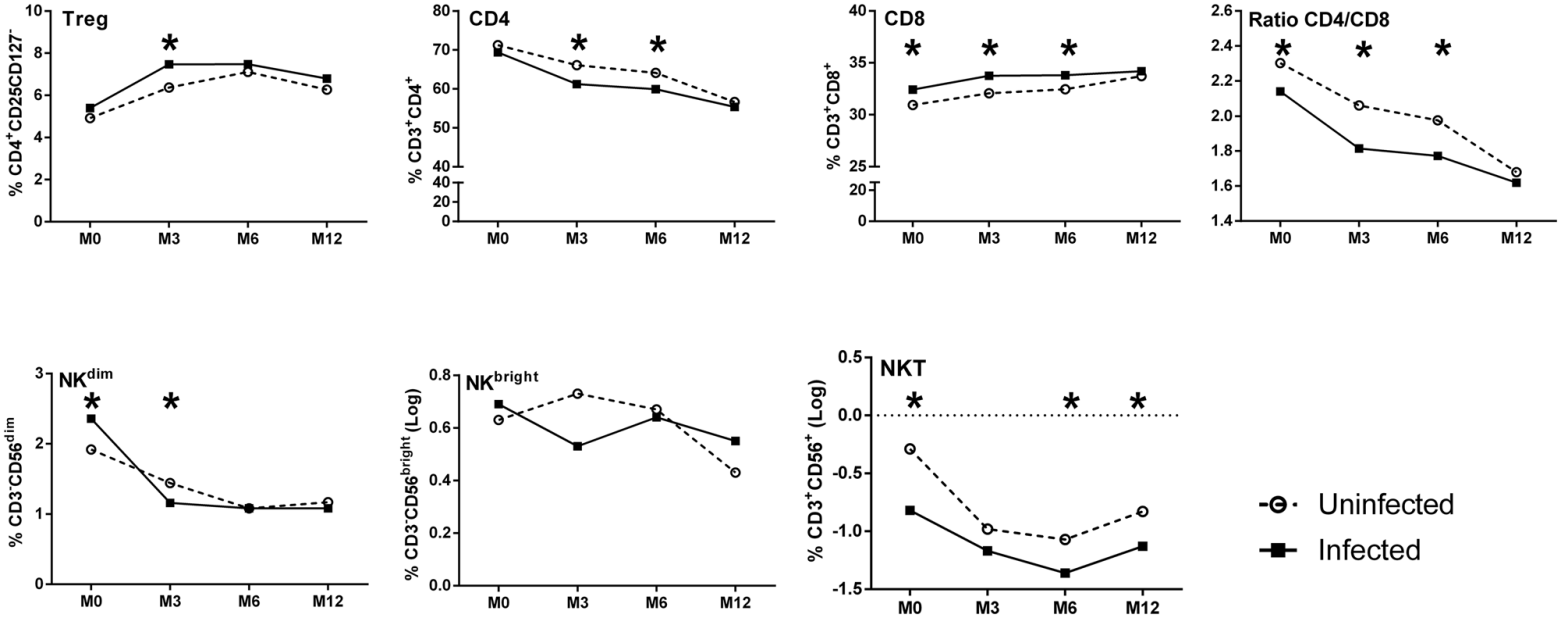
Adjusted profiles of lymphocyte frequencies in infants during the first year of life according to *P*. *falciparum* exposure *in utero*. Values are derived from residuals in the multivariate LMM model. The statistical significance of differences indicated refers to those presented in Tables 2 and 3. *, p <0.05.

The results of multivariate analyses of the effects of maternal infection at delivery are illustrated in Tables 2 and 3 with a graphical depiction in Fig 4 of an adjusted model, based on the multivariate LMM analysis. The significantly increased frequencies of CD8^+^ cells in infants at birth, 3 and 6 months of age as well as increased proportions of Treg in infants at 3 months of age were confirmed in the multivariate analyses (Fig 4). In addition, the significantly reduced frequencies of CD4^+^ cells in infants at 3 and 6 months of age and lower NKT cell frequencies at birth, 6 and 12 months of age (Fig 4) were also confirmed. A significant increase in the frequency of infant NK^dim^ cells was shown at birth, but significantly lower frequencies were present at 3 months of age (Fig 4).

In summary, maternal infection at delivery had a significant effect on the profile of immune cells of the infant, with increased frequencies of Treg and CD8^+^ cells and a reduced percentage of CD4^+^ and NKT cells. Accordingly, the CD4/CD8 ratio was also reduced over time in infants born to mothers that were infected close to or at delivery.

### *P*. *falciparum* infection in infants impacts on the immune cell profile

We next evaluated the effect of *P*. *falciparum* infections in infants on their immune cell profile during the first year of life. (S1 Table & Table 4). We observed that infants infected before 3 months of age had higher frequencies of CD4^+^ and lower frequencies of CD8^+^ cells than those not infected in the same period. In addition, infants infected between 3 and 12 months of age exibited a lower frequency of Treg than those uninfected in the same period as well as a lower frequency of Treg expressing CD25^high^ in infants infected between 3 and 6 months of age by univariate analysis (S1 Table). In the logistic mixed model (Table 4), the impact of infant *P*. *falciparum* infection on their T-/NK-cell frequencies was examined prospectively according to their infection histories 3 months prior to blood sampling at 3, 6 and 12 months. At 3 months, those infected previously had more CD4^+^ and fewer CD8^+^ cells. At 6 months, infants infected previously had lower relative values of FoxP3 in Treg and in TregCD25^high^. At 12 months infants infected previously had a tendency towards higher frequencies of NKT cells but lower frequencies of Treg (Table 4).

**Table 4:** A logistic mixed model to evaluate the impact of infant *P*. *falciparum* infection on the frequencies of circulating lymphocyte sub-types.

		Impact of infant malaria between 0-3M on cellular responses at 3M		Impact of infant malaria between 3-6M on cellular responses at 6M		Impact of infant malaria between 6–12 M on cellular responses at 12M	
		Estimate (SD)	p-value	Estimate (SD)	p-value	Estimate (SD)	p-value
Treg	Infected	-0.88 (0.9)	0.33	-0.59 (0.73)	0.422	-0.91 (0.4)	0.024
Teff	Infected	0.26 (0.65)	0.685	-0.38 (0.55)	0.491	-0.44 (0.42)	0.295
Treg CD25^high^	Infected	0.03 (0.07)	0.637	-0.07 (0.05)	0.198	-0.02 (0.03)	0.576
Treg/Teff ratio	Infected	-0.85 (0.57)	0.136	0.03 (0.25)	0.896	-0.31 (0.2)	0.127
FoxP3 RV in Treg	Infected	-0.18 (0.31)	0.575	-0.34 (0.12)	0.006	0.06 (0.07)	0.402
FoxP3 RV in Teff	Infected	-0.04 (0.15)	0.765	-0.13 (0.13)	0.299	-0.08 (0.08)	0.325
FoxP3 RV in Treg CD25^high^	Infected	-0.21 (0.91)	0.819	-0.89 (0.32)	0.006	0.14 (0.20)	0.482
CD4^+^	Infected	9.66 (4.12)	0.02	3.60 (2.83)	0.21	-1.91 (2.27)	0.4
CD8^+^	Infected	-9.58 (3.66)	0.01	-3.34 (2.24)	0.14	1.37 (1.89)	0.47
NK^dim^	Infected	-1.92 (1.56)	0.22	0.26 (0.55)	0.64	0.33 (0.51)	0.52
NK^bright^	Infected	0.37 (0.25)	0.15	-0.03 (0.19)	0.87	0.01 (0.08)	0.91
NKT	Infected	0.00 (0.18)	0.99	0.08 (0.16)	0.61	0.14 (0.07)	0.06

Estimate: Positive/negative coefficients indicate cell subsets frequencies above/below control (uninfected) levels. All the data were adjusted on *P*. *falciparum* infection history of mother, gravidity, infant age, low birth weight.SD: standard deviation

### Immunological predictors of the occurrence of *P*. *falciparum* in infants

Finally, we wanted to determine whether the varying frequencies of the cell populations we investigated were associated with a higher probability of *P*. *falciparum* infection in infancy.

Firstly, we assessed whether cord blood cell frequencies were predictive of *P*. *falciparum* infection during infancy. In univariate analysis, lower cord blood Teff but higher CD4^+^ cell frequencies were associated with an increased risk of *P*. *falciparum* infection during the first year of life (Table 5). In multivariate analysis, only the association with a significantly lower frequency of Teff remained.

**Table 5:** T and NK cell cord blood frequencies as immunological predictors of malaria in infants.

	Univariate			Multivariate		
	OR	IC 95%	p-value	Ajusted OR	IC 95%	p-value
Treg	0.90	[0.66; 1.22]	ns	1.14	[0.80; 1.61]	0.47
Teff	0.55	[0.31; 1.00]	0.04	0.49	[0.26; 0.92]	0.02
CD4^+^	1.07	[1.00; 1.15]	0.04	1.03	[0.89; 1.18]	0.69
CD8^+^	0.94	[0.87; 1.01]	0.09	0.97	[0.83; 1.13]	0.68
NK (NK^dim^ + NK^bright^)*	0.98	[0.92; 1.05]	ns	0.99	[0.92; 1.06]	0.73

Data were adjusted on the *P*. *falciparum* infection history of the mother, gravidity, infant age, low birth weight.

*Results cumulate both NK^dim^ and NK^bright^ to avoid introducing two variables that are highly correlated into the model.

We next examined the impact of cell frequencies at specific time-points on the occurrence of *P*. *falciparum* infection in the ensuing months of infancy. Univariate analysis showed that higher proportions of Treg, Teff and CD8^+^ cells and lower proportions of CD4^+^ and NK cells were significantly associated with an increased risk of *P*. *falciparum* infection in the first year of life. Multivariate analysis showed that lower proportions of Teff, CD4^+^, NK cells and higher proportions of CD8^+^ cells were significantly associated with an increased risk of developing *P*. *falciparum* infection during the first year of life (Table 6).

**Table 6:** T-/NK-cell frequencies and the occurrence of malaria during the first 12 months of life.

		Univariate		Multivariate Model		
	OR	IC 95%	p-value	Adjusted OR	IC 95%	p-value
Treg	1.20	[1.08; 1.33]	<0.001			
Teff	1.29	[1.18; 1.42]	<0.001	0.85	[0.74; 0.97]	<0.001
CD4^+^	0.95	[0.93; 0.97]	<0.001			
CD8^+^	1.05	[1.02; 1.08]	<0.001	1.07	[1.02; 1.11]	<0.01
NK (NK^dim^ + NK^bright^)*	0.87	[0.83; 0.91]	<0.001	0.79	[0.73; 0.87]	0.02

Data were adjusted on the *P*. *falciparum* infection history of the mother, gravidity, infant age, low birth weight

*Results cumulate both NK^dim^ and NK^bright^ to avoid introducing two variables that are highly correlated into the model.

## Discussion

The characterization of factors associated with increased susceptibility of infants to *P*. *falciparum* infection during the first year of life is a priority in the development of a vaccine against malaria. Here, we performed a longitudinal study of immune cell profiles in the first year of life of a large cohort of infants born to mothers with varying histories of infection during pregnancy.

We observed significant age-related changes in the frequencies of CD4^+^, CD8^+^, Treg, Teff, NK and NKT cells from birth to 12 months of age. Those age-related changes were thus taken into consideration in multivariate analyses assessing the impact of *P*. *falciparum* exposure *in utero* or infection during the first year of life on infants' immune cell frequencies.

It was, firstly, notable that the CD4^+^ cell frequency was highest at birth and then declined over the ensuing 12 months whilst the reverse was seen for CD8^+^ cell frequencies. The decline of CD4^+^ cell frequency in relation to age contrasts with the stable numbers reported by some [24], but is consistent with the decline seen in older-age children (1–13 years) reported by others [48, 49]. Similarly, the increasing CD8^+^ cell frequency during the first 12 months of life is consistent with some but not all previous reports [24, 49]. The disparities in reported findings may simply reflect heterogeneity of the different study populations. Mechnistically, declining numbers of CD4^+^ T cells during the first year of life may be explained by apoptosis [50] an age-dependent phenomenon, as cord blood lymphocytes are less sensitive to TNF-α-induced apoptosis than aged T cells [51]. Alternatively or additionally, it could reflect migration of Treg out of the circulation to the gut, which is the primary site of Treg stimulation in responses to exogenous antigens during the first 18 month of life through specific homing receptors expressed specifically at birth [52]. Also, it has been reported that neonatal CD8^+^ T cells proliferate more than CD4^+^ T cells as CD8^+^ T cells are more sensitive to IL-7 than CD4^+^ T cells, a process that decreases during the first years of life [53–55].

The fact that the frequencies of Treg and Teff increased from birth to 12 months of age—in marked contrast to the overall decline of CD4^+^ T cells, it should be noted—is likely an indication of the development of infant immunity through the first year of life. The increased Treg frequency and increased relative expression of FoxP3 in the CD25^high^ subpopulation of Treg during the first year of life are in line with studies reporting increased proportions of FoxP3 T cells in peripheral blood soon after birth compared to cord blood [52, 56]. Treg maturation and their suppressive function is FoxP3-dependent. It has been demonstrated that cord blood cells express lower levels of Foxp3 compared to adult cells and that this contributes to the immature nature of neonatal immunity [57–59].

The two functionally distinct NK-cell subpopulations, cytotoxic CD56^dim^ NK cells and cytokine-producing CD56^bright^ NK cells, showed contrasting profiles, with a gradual decrease of the NK CD56^dim^ and increasing NK CD56^bright^ during the first year of life. Others have reported similar findings [24]. NK cells play a major role in innate immune responses that are relatively more important in early life when adaptive immunity has yet to develop, possibly explaining the high frequency of cytotoxic NK^dim^ cells present at birth. NKT cells are present at comparable frequencies in cord blood and adult peripheral blood mononuclear cells [60]. We have not found data other than our own concerning the gradual decrease in NKT frequencies from birth to 6 months of age. NKT cells *in utero* are unique in acquiring a memory-activated phenotype through a contact with a natural ligand [42], suggesting that these NKT cells already exert an important immune regulatory function before birth and in self tolerance. Decreased post-natal NKT frequencies may be related to the overall change in the frequency distibution of different lymphocyte phenotypes.

In the context of the influence of maternal infection with *P*. *falciparum* on neonatal profiles, perhaps the most notable finding concerns the changes in CD4^+^ and CD8^+^ cells. Children born to mothers infected at delivery exhibited a skewed profile of CD4^+^ and CD8^+^ cell subsets, with an increased frequency of CD8^+^ and a reduced frequency of CD4^+^ cells at 3 and 6 months of age and consequently a continuous decline in the CD4/CD8 ratio between birth and 12 months of age. These patterns, as indicated earlier, are superimposed on the age-related trends—going in the same direction—for these same two cell types, suggesting possible additive effects of age and maternal infection on CD4^+^ and CD8^+^ cell profiles during infancy. CD4^+^ T cells are considered essential triggers of early and effective NK-cell IFN-γ responses upon contact with *P*. *falciparum*-infected erythrocytes [61]. They are, furthermore, required for generation and maintenance of protective antibody-mediated responses to *P*. *falciparum* and other pathogens [62]. Excessive loss of such cells during infancy may therefore play a pivotal role in the enhanced susceptibility to infection suffered by those born to mothers with placental infection.

The Treg and Teff frequencies were similar in cord blood of newborns born to mothers with *P*. *falciparum* infection compared to those born to uninfected mothers, again consistent with some previous studies [22, 63] but not with others [20, 64, 65]. *In utero* exposure to *P*. *falciparum* at delivery was nevertheless associated with significantly higher frequencies of Treg at three months of age. Persistence of Treg following *in utero* contact with non-self antigens is a reported phenomenon [30, 66]. Several studies have shown that *ex vivo* stimulation of cord blood cells with *P*. *falciparum* blood stage antigens reveals populations of CD4^+^ T cells with a Treg phenotype [67]. Such cells, in cord blood of offspring born to women with placental malaria, have been shown to produce more IL-10 compared to cells of those born to mothers without placental malaria [64, 65]. Compatible with these findings, others have shown that a soluble extract of *P*. *falciparum-*infected red blood cells induces the differentiation of polyclonally-activated Treg CD4^+^ T cells with strong suppressive activity, and that this activation was mediated by membrane bound TGF-β on the Treg cells leading to immune evasion and reduced pro-inflammatory responses [29].

Resource constraints meant that we were unable to assess the functional characteristics of the different T cell subsets that we quantified, but we hypothesize that higher numbers of Treg in infants born to mothers infected at delivery would be associated with enhanced immunoregulatory activity and with concomitantly reduced pro-inflammatory responses. Such a profile would likely contribute to the increased susceptibility to infections observed in children born to infected mothers [13–15, 20]. Treg induced during infection are thought to limit the magnitude of subsequent parasite-specific IFN-γ responses [68]. Prenatal exposure to plasmodial blood-stage antigens induces Treg that, in some newborns, primarily suppress Th1- type recall responses [2, 20]. Thus, the persistence of such Treg post-natally may affect the susceptibility of children both to *P*.*falciparum* and to other infections. We did not observe any alteration of the relative expression of FoxP3 in Treg or Teff. It could have been more informative to determine the expression of other functional markers such as CTLA-4 and/or TNFRII. It is known that Treg can dampen NK-cell activation/ proliferation and cytotoxic activity [69, 70] as well as NKT cell proliferation and cytokine production [39]. In line with these observations, and in association with the increased Treg frequency in infants born to mothers infected at delivery, we found a reduced frequency of circulating NKT cells in infants of mothers infected with *P*. *falciparum* close to or at delivery as compared to the other group at birth. Further, maternal infection close to or at delivery was associated with an increased frequency of infant NK^dim^ cells at birth, and to lower frequencies of these cells at 3 months of age. These observations are suggestive of an early inflammatory response being needed to protect against plasmodial parasites. Relatively diminished pro-inflammatory activity—as reflected by cord plasma cytokine profiles—is associated with more severe clinical outcomes of infection with *P*. *falciparum* during infancy [71, 72]. Assessing plasma cytokine profiles in our cohort of infants is thus of obvious interest in the context of the findings we present here as well as those we have previously reported [73].

Infection with *P*. *falciparum* in infancy also altered the immune cell profile, with infants infected before 3 months of age having a higher frequency of CD4^+^ T cells and a lower frequency of CD8^+^ T cells. It should be stressed that this, again, is a pattern superimposed on what is, in this case, the markedly contrasting background of an overall age-related decline in CD4^+^ cells and an increase in CD8^+^ cells, a pattern that, as was noted earlier, placental infection at delivery further exacerbated. Again, we have no specific functional data on which to base any interpretation of this observation. It is nevertheless plausible to suppose that any infection-induced expansion of a population of CD4^+^ T cells in such infants will primarily comprise atypical, transitional cells that have not undergone full maturation. Theory suggests that such effector-type cells induced in infancy remain in a state of anergy with little if any functional anti-pathogen activity [74].

Reduced Treg frequencies were observed in infants who were infected between 6 and 12 months of age, and the relative expression of FoxP3 in the CD4^+^CD25^+^ and CD25^high^ CD4^+^ subpopulation was reduced in infants who were infected between 3 and 6 months of age. These findings contrast with the increase in number of Treg in the peripheral blood of older children and adults during *P*. *falciparum* infections [26–29]. The data we present here comprise observations after the infection event, however, and may thus simply reflect the physiological contraction of pathogen-induced responses following clinical recovery and clearance of the parasite.

One of the major limitations of our study concerns the absence of data with respect to co-infections such as, for example, chronic maternal helminth infections. Such infections are known to affect anti-plasmodial immune responses [75–77]. Furthermore, contact *in utero* with maternally-derived filariasis antigens reportedly modifies cord blood cell responses such that they display a predominantly anti-inflammatory profile [78]. On the other hand, the prevalence of (soil-transmitted) helminth infections in the first 12 months of life is likely less than 10% [79], and thus any possible influence on the lymphocyte profiles we observed here is likely to have been minimal.

In conclusion, we have found that maternal infection with *P*. *falciparum* at delivery, but not earlier in pregnancy profoundly affects the immune cell profile of their babies, with, in some cases, effects lasting at least 1 year. Cells of both the innate and adaptive arms of the immune system are affected. These findings echo our observations on TLR-mediated innate immune responses of the same group of infants [73]. Infection at delivery, but not earlier in pregnancy, was associated with significantly higher TLR3-mediated IL-6 and IL-10 responses in the first 3 months of life, and with significantly higher TLR3-/TLR7/8-/TLR9-mediated TNF-α and TLR9-mediated IL-10 responses at 6 or 12 months of age. Whether those alterations, along with the maternal infection-related relative increase in CD8^+^ cells and relative decrease in CD4^+^ cells in the post-natal period that we report here, have a direct bearing on infants' immune response to infection remains an open question. Plausibly, such changes could form the basis for an explanation of the increased susceptibility to malaria of infants born to mothers with infections at delivery, but proof of causality awaits further study.

## Supporting Information

S1 Fig The gating strategies for Treg (CD4^+^CD25^+^CD127^-^) and Teff (CD4^+^CD25^+^CD127^+^). Cell frequencies were determined as a percentage from the whole lymphocyte population, and relative FoxP3 expression level determined as a function of FoxP3 expresssion by naïve CD4^+^ T cells (CD4^+^CD25^-^).
(TIFF)

S1 Table Univariate analysis of alterations in circulating T- and NK-cell subset frequencies in cord/infant blood as a function of *P*. *falciparum* infection detected either in the mother at delivery or during infancy (DOCX)

## Abbreviations

ANV: antenatal visit
CERPAGE: research center for pregnancy associated malaria and children
HIV: human immunodeficiency virus
IRD: Research Institute for Development
LMM: linear mixed models
PAM: pregnancy associated malaria
P. falciparum: *Plasmodium falciparum*
RDT: rapid diagnostic test
STOPPAM: Strategies TO Prevent Pregnancy Associated Malaria
TBS: thick blood smear
