# Supplementary figures and images for: Infants' Peripheral Blood Lymphocyte Composition Reflects Both Maternal and Post-Natal Infection with Plasmodium falciparum
Source: PLoS One. 2015 Nov 18;10(11):e0139606. doi: 10.1371/journal.pone.0139606 (PMC4651557; doi:10.1371/journal.pone.0139606)

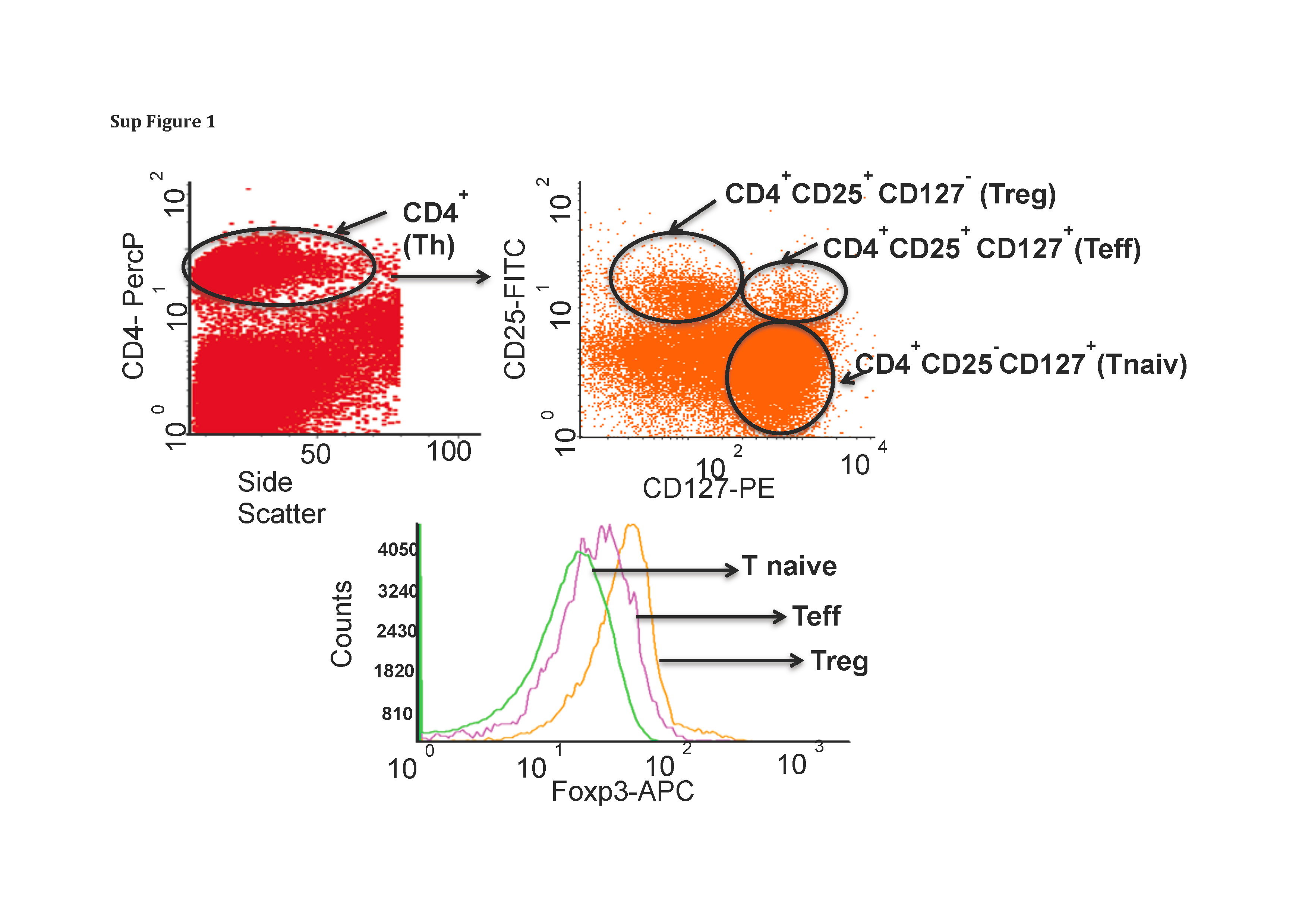

Supplement: S1 Fig — Cell frequencies were determined as a percentage from the whole lymphocyte population, and relative FoxP3 expression level determined as a function of FoxP3 expresssion by naïve CD4+ T cells (CD4+CD25-). (TIFF) [file pone.0139606.s001.tiff]
