# Supplementary material for: Infants' Peripheral Blood Lymphocyte Composition Reflects Both Maternal and Post-Natal Infection with Plasmodium falciparum
Source: PLoS One. 2015 Nov 18;10(11):e0139606. doi: 10.1371/journal.pone.0139606 (PMC4651557; doi:10.1371/journal.pone.0139606)
Supplement: S1 Table — (DOCX) [file pone.0139606.s002.docx]

Supplementary Table 1: Univariate analysis of alterations in circulating T- and NK-cell subset frequencies in cord/infant blood as a function of *P. falciparum* infection detected either in the mother at delivery or during infancy

|  |  | Treg |  | Teff |  | Treg (CD25^high^) |  | Treg/Teff |  | RV FoxP3  in Treg |  | RV FoxP3  in Teff |  | RV FoxP3  inTreg (CD25^high^) |  |
| --- | --- | --- | --- | --- | --- | --- | --- | --- | --- | --- | --- | --- | --- | --- | --- |
|  |  | Coef (SD) ^A^ | p | Coef (SD) | p | Coef (SD) | p | Coef (SD) | p | Coef (SD) | p | Coef (SD) | p | Coef (SD) | p |
| *P. falciparum* mother | at delivery ^B^ | 0.63 (0.28) | <0.05 | 0.04 (0.20) | ns | 0.02 (0.02) | ns | 0.18 (0.17) | ns | 0.09 (0.06) | ns | -0.02 (0.04) | ns | 0.23 (0.15) | ns |
|  |  |  |  |  |  |  |  |  |  |  |  |  |  |  |  |
| *P. falciparum* infant ^C^ | M0-M3 | -0.58 (0.63) | ns | 0.26 (0.44) | ns | -0.06 (0.05) | ns | -0.54 (0.39) | ns | -0.20 (0.15) | ns | -0.08 (0.08) | ns | -0.28 (0.33) | ns |
|  | M4-M6 | -0.69 (0.36) | 0.05 | -0.43 (0.26) | 0.08 | -0.06 (0.03) | <0.05 | 0.13 (0.23) | ns | 0.02 (0.09) | ns | -0.07 (0.05) | ns | 0.07 (0.20) | ns |
|  | M7-M12 | -0.59 (0.26) | <0.05 | -0.31 (0.19) | 0.08 | -0.02 (0.02) | ns | -0.07 (0.16) | ns | 0.02 (0.06) | ns | 0.008 (0.03) | ns | -0.09 (0.14) | ns |

|  |  | CD4+ |  | CD8+ |  | NKT |  | NK ^dim^ |  | NK^bright^ |  |
| --- | --- | --- | --- | --- | --- | --- | --- | --- | --- | --- | --- |
|  |  | Coef (SD) | p | Coef (SD) | p | Coef (SD) | p | Coef (SD) | p | Coef (SD) | p |
| *P. falciparum* mother | at delivery | -3.20 (1.30) | <0.05 | 3.19 (1.11) | <0.01 | -0.23 (0.08) | <0.05 | 0.86 (0.50) | 0.07 | -0.007 (0.07) | ns |
|  |  |  |  |  |  |  |  |  |  |  |  |
| *P. falciparum* infant | M0-M3 | 7.44 (2.69) | <0.05 | -7.04 (2.38) | <0.01 | -0.05 (0.18) | ns | -1.25 (1.04) | ns | 0.15 (0.14) | ns |
|  | M4-M6 | 2.51 (1.94) | ns | -2.83 (1.67) | 0.08 | 0.06 (0.13) | ns | -0.49 (0.75) | ns | 0.05 (0.10) | ns |
|  | M7-M12 | -0.81 (1.38) | ns | 0.72 (1.19) | ns | 0.002 (0.09) | ns | -0.20(0.53) | ns | 0.03 (0.07) | ns |

^A^ positive/negative coefficients indicate cell subsets frequencies above/ below control (uninfected) levels; SD: standard deviation; ^B^ denotes the influence of infection at delivery or in the time-period < 10 days prior to delivery on neonatal/infant cell subsets frequencies in the first 12 months of life; ^C^ denotes the influence of infection during different periods of early life on cell subsets frequencies over the whole 12 month period ; M0: cord blood, M3, M6, M12: blood drawn at 3, 6 & 12 months of age
